# Differences in characteristics and outcomes of older patients hospitalized for COVID-19 after introduction of vaccination

**DOI:** 10.1007/s41999-024-01002-0

**Published:** 2024-06-11

**Authors:** Rosalinde A. L. Smits, Bas F. M. van Raaij, Stella Trompet, Carolien M. J. van der Linden, Jessica M. van der Bol, Steffy W. M. Jansen, Harmke A. Polinder-Bos, Hanna C. Willems, Esther M. M. van de Glind, Julia Minnema, Lisanne Tap, Simon P. Mooijaart

**Affiliations:** 1 Section Gerontology and Geriatrics, Department of Internal Medicine, Leiden University Medical Centre, Albinusdreef 2, 2333 ZA Leiden, The Netherlands; 2 Department of Geriatrics, Catharina Hospital, Eindhoven, The Netherlands; 3 Department of Geriatric Medicine, Reinier de Graaf Hospital, Delft, The Netherlands; 4 Department of Internal Medicine, Erasmus MC, University Medical Centre, Rotterdam, The Netherlands; 5 Section Geriatrics, Department of Internal Medicine, Amsterdam University Medical Centre, Location AMC, Amsterdam, The Netherlands; 6 Department of Geriatrics, Alrijne Hospital, Leiderdorp, The Netherlands; 7 LUMC Centre for Medicine for Older People, Leiden University Medical Centre, Leiden, The Netherlands

**Keywords:** Vaccination, COVID-19, In-hospital mortality, Frailty, Older patient

## Abstract

**Purpose:**

The aim of the present study was to investigate characteristics and outcomes in vaccinated and unvaccinated older patients hospitalized for COVID-19 infection.

**Methods:**

A retrospective multicentre cohort study among patients aged ≥70 years hospitalized for COVID-19 infection.

**Results:**

263 vaccinated and 82 unvaccinated patients were included. Vaccinated patients were older (median age 79 vs. 76 years; p < 0.001), more patients were male (66.2% vs. 53.7%; p = 0.040), had more comorbidities [median Charlson Comorbidity Index (CCI) 2 vs. 1; p 0.016] and were frailer [Clinical Frailty Scale (CFS) ≥ 4 68% vs. 49%; p 0.015]. Vaccinated patients were admitted earlier after symptom onset (median 5 days vs. 7 days) but were equally ill at time of hospital admission. After correction for frailty, comorbidity and disease severity, risk of in-hospital mortality was three times lower for vaccinated patients (HR 0.30 95% CI 0.16–0.56; p < 0.001) compared to unvaccinated patients.

**Conclusion:**

Vaccinated patients had lower risk of in-hospital mortality than unvaccinated patients with COVID-19 infection. These findings suggest that vaccinated patients benefit from the protective effect of the vaccine against death during hospital stay, outweighing the increased mortality risk that is associated with older age, greater frailty and more numerous comorbidities. This could be an encouragement for older people to receive age-appropriate vaccines, although no definite conclusions can be drawn for this was no intervention study.

## Key summary points

**Aim:**

The aim of the present study was to investigate differences in characteristics and outcomes between vaccinated and unvaccinated older patients hospitalized for COVID-19 infection.

**Findings:**

Compared to older unvaccinated patients hospitalized for COVID-19, vaccinated patients were frailer, had more comorbidities and, independent of these factors, a three times lower risk for in-hospital mortality.

**Message:**

Although being frailer, vaccinated older patients have a lower risk of in-hospital mortality than unvaccinated patients.

## Introduction

Since the introduction of COVID-19 vaccines at the end of 2020, the beneficial effect of vaccination on risk of hospital admission, in-hospital outcomes and mortality has been proven [1, 2]. However, it is not yet known if older vaccinated COVID-19 patients differ from older unvaccinated COVID-19 patients in terms of frailty, comorbidity and disease severity at the moment of hospitalization. The association of vaccination with mortality, specifically for frail older hospitalized people, has been studied recently in Taiwan in a cohort of older hospitalised patients with COVID-19 infection in 2022, after introduction of vaccination. The results showed no significant effect of vaccination on in-hospital mortality in logistic regression analysis, but mortality was low compared to mortality in the beginning of the pandemic [3].

In The Netherlands, 89% to 93% of people aged 70 years and older were vaccinated at least once in the first year of the vaccination campaign in 2021 [4]. Older people with frailty and comorbidities are more likely to be vaccinated, since nursing home practitioners, general practitioners and national campaigns of the RIVM (National Institute for Public Health and the Environment) [4] actively stimulated vaccination in this group of people. Potentially, vaccination may have affected the selection of older people with COVID-19 that needed hospitalization and their disease course during hospitalization, but this has not been quantified yet. In case more frail older people are hospitalized for COVID-19 infection, pro-active geriatric advance care planning, aimed for example towards early rehabilitation, may be needed.

Therefore, the aim of the present study was to investigate characteristics and outcomes in vaccinated and unvaccinated older patients hospitalized for COVID-19 infection in The Netherlands.

## Methods

### Study design

This was a retrospective multicentre cohort study among patients aged 70 years and older who were hospitalized for COVID-19 infection in time period September 1st until December 31st 2021 in The Netherlands. Data were collected from 5 Dutch hospitals: Alrijne Hospital (Leiden), Catharina Hospital (Eindhoven), Erasmus MC University Medical Centre (Rotterdam), Reinier de Graaf Hospital (Delft) and Leiden University Medical Centre (Leiden). The medical ethics committees of all hospitals waived the necessity for formal approval of the study, as data collection followed routine practice. Further details of the study design can be found in earlier publications [5, 6].

### Setting

#### Organization of healthcare in the Netherlands

In the Netherlands, basic health insurance is mandatory and covers primary care from general practitioners (GPs) and hospital care. In case of a medical emergency, patients can contact their GP, visit the GP out-of-hours service, call for an ambulance or go to the Emergency Department (ED).

Entry in the Emergency Department follows after selection by these pre-hospital professionals.

#### COVID-19 variants, medication regimen and vaccination

In the third pandemic wave (fall 2021) the Delta variant was dominant in Europe and found in 100% of test samples the Netherlands until December 2021, when the first cases of OmicronBA.1 have been detected [7]. During hospitalization most patients received dexamethasone solely or combined with tocilizumab or sarilumab. Immunocompromised patients were tested for antibodies and received casirivimab/imdevimab, according to the Dutch treatment guideline based on the WHO international guideline [8].

In The Netherlands, the COVID-19 vaccination program started January 6th 2021 with the vaccination of nursing home residents and community-dwelling people born before 1931. In the following months, all Dutch residents were invited to be vaccinated. All patients included in our study have had the opportunity to be fully vaccinated prior to the inclusion period. At September 1st 2021, of all community-dwelling people 70 years and older, 89 to 93% had received at least one COVID-19 vaccination [4].

### Study participants

The inclusion criteria were patients aged 70 years and older, hospitalized between September 1st and December 31st 2021 for a PCR test confirmed COVID-19 infection with known COVID-19 vaccine status. Patients diagnosed with COVID-19 infection in the hospital during admission for another illness were excluded, defined as a positive PCR test more than 1 day after admission. Furthermore, patients who were transferred between hospitals were excluded, because baseline and outcome data were incomplete for these patients.

### Outcomes

The main outcome of this study was in-hospital mortality. Other outcomes of interest were hospital length of stay in days and discharge destination (home, rehabilitation centre or other destination).

### Data collection

Data were collected from the patient’s electronic healthcare records. We collected demographic data on age, sex and living situation (at home or institutionalized). The Charlson Comorbidity Index (CCI) was used to assess the presence of comorbidities [9].

Geriatric parameters were routinely collected with the Dutch National Safety Management System (VMS) [10]. This risk assessment tool was used at hospital admission for all patients aged ≥70 years. The instrument consists of thirteen questions about four domains: physical impairment, falls, delirium and malnutrition. Physical impairment was evaluated using the Katz Activities of Daily Living (ADL) Index [11]. A score ≥2 is defined as risk of physical impairment. A fall in the last six months is defined as risk of falling. One or more positive answers to questions on memory problems, the need for help with self-care in the last 24 h and previously experienced confusion is defined as a risk for delirium. For evaluation on malnutrition the instruments Short Nutritional Assessment Questionnaire (SNAQ) or Malnutrition Universal Screening Tool (MUST) were used. A score of SNAQ ≥ 3 or MUST ≥ 2 is defined as a risk of malnutrition [12].

Frailty was assessed with the Clinical Frailty Score (CFS) [13]. The CFS was preferably prospectively determined at hospital admission according to the implemented NICE guidelines (most of the times by a geriatrics nurse) [14]. If not prospectively assigned, the CFS was determined retrospectively, based on available chart data (which included the geriatric parameters from the VMS) and was scored by a researcher trained by a geriatrician or internist-geriatrician. The CFS can be categorized in three groups: fit (CFS 1–3), pre-frail (CFS 4–5) and frail (CFS 6–9) and considered the level of frailty two weeks prior to onset of COVID-19 infection.

We defined a patient vaccinated in case he or she received at least one COVID-19 vaccination dose prior to hospital admission. This was a self-reported variable.

Data were collected using Castor Electronic Data Capture (2022).

### Statistical analyses

Patients were categorized into vaccinated and unvaccinated groups. Patient characteristics and disease severity indicators were presented as continuous and categorical variables.

In the descriptive analyses continuous data were presented as means and standard deviation (SD) if normally distributed, and as medians and interquartile range (IQR) if skewed. Categorical data were presented as absolute values and percentages. Differences in patient characteristics, disease severity indicators and outcomes were assessed using unpaired T-tests for normally distributed data, Mann Whitney U test for skewed data and χ^2^ test for categorical data. A univariable Cox regression analysis was performed with the number of admission days as time, discharge as censoring and in-hospital mortality as outcome to investigate the relation between patient characteristics and in-hospital mortality. We also performed a Cox regression multivariable analysis including different models, to investigate a possible independent effect of vaccination on in-hospital mortality. The models contain variables known to be associated with in-hospital mortality: demographics (age, male sex, living at home), disease severity (respiratory rate) and frailty and comorbidity indicators (CFS, CCI). Results are presented as hazard ratios (HRs) with 95% confidence intervals (95% CI). A p-value < 0.05 was considered statistically significant.

Statistical analyses were performed using IBM SPSS Statistics for Windows, version 25 (IBM Corp., Armonk, N.Y., USA).

## Results

**Fig. 1:**
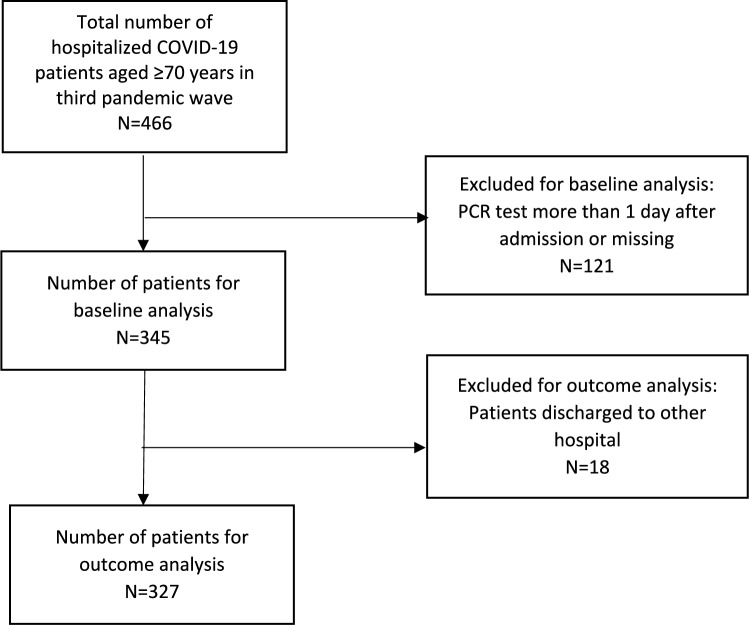
Flowchart

Figure 1 shows the flowchart of included patients. A total of 466 patients were identified. We excluded 121 patients that had been tested for COVID-19 more than 1 day after admission, resulting in 345 patients included for baseline analysis. Additionally, 15 patients were discharged to another hospital and outcome data were not available, resulting in 327 patients eligible for outcome analysis.

Baseline characteristics of vaccinated and unvaccinated patients are displayed in Table 1. A total of 82 unvaccinated (23.8%) and 263 vaccinated (76.2%) patients were admitted. Compared to unvaccinated patients, vaccinated patients had a higher median age [79 years (IQR 75–85) vs. 76 years (IQR 73–80); p < 0.001], were more often male (66.2% vs. 53.7%; p = 0.040), were more often (pre-)frail (CFS ≥ 4 67.9% vs. 49.3%; p = 0.006) and were less frequently living at home (92.5% vs. 98.7%: p = 0.048). In addition, compared to unvaccinated patients, vaccinated patients had more comorbidities [median CCI 2 (IQR 1–3) vs. 1 (IQR 0–2); p = 0.016] and were hospitalized in an earlier stage of disease [symptomatic days until admission 5 (IQR 2–8) vs. 7 (IQR 4–10); p = 0.008]. Disease severity indicators at time of admission (temperature, respiratory rate, oxygen amount needed and CRP) were similar in both groups.

**Table 1:** Baseline characteristics for older hospitalized COVID-19 patients stratified by vaccination status

	Total group	Unvaccinated	Vaccinated	p-value
	N = 345	N = 82	N = 263	
Demographics				
Age (years), median (IQR)	79 (74–84)	76 (73–80)	79 (75–85)	<0.001
Male sex, n (%)	218 (63.2)	44 (53.7)	174 (66.2)	0.040
Living at home, n (%)	310 (93.9)	75 (98.7)	235 (92.5)	0.048
Comorbidity				
Charlson Comorbidity Index, median (IQR)	2 (1–3)	1 (0–2)	2 (1–3)	0.016
History of chronic lung disease^a^, n (%)	107 (31.0)	17 (20.7)	90 (34.2)	0.042
History of dementia, n (%)	18 (5.2)	3 (3.7)	15 (5.7)	0.467
Geriatric measurements				
Katz ADL score, median (IQR)	0 (0–2)	0 (0–2)	0 (0–2)	0.279
Risk of physical impairment^b^, n (%)	92 (32.7)	17 (27.4)	75 (34.2)	0.312
Risk of falling, n (%)	66 (23.7)	11 (13.4)	55 (20.9)	0.214
Risk of delirium, n (%)	101 (37.8)	15 (26.8)	86 (40.8)	0.055
Risk of malnutrition, n (%)	34 (14.0)	8 (14.5)	26 (13.9)	0.904
Clinical Frailty Scale, n (%)				0.015
1–3 (fit)	105 (36.5)	34 (50.7)	71 (32.1)	
4–5 (pre-frail)	97 (33.7)	15 (22.4)	82 (37.1)	
6–9 (frail)	86 (29.9)	18 (26.9)	68 (30.8)	
Disease severity indicators				
Duration of symptoms until admission (days), median (IQR)	6 (3–9)	7 (4–10)	5 (2–8)	0.008
Body temperature (°C), mean (SD)	37.7 (1.02)	37.8 (1.06)	37.6 (1.01)	0.198
Hospital length of stay (days), median (IQR)	7 (4–12)	7 (3–14)	7 (4–12)	0.810
Respiratory rate (breaths/min), median (IQR)	22 (18–27)	22 (18–27)	22 (18–27)	0.371
Oxygen amount needed (L/min), median (IQR)	3 (1–6)	3 (1–12)	2 (1–5)	0.123
No oxygen amount needed at admission, n (%)	67 (21.2)	12 (16.0)	55 (22.8)	0.207
C-reactive protein (mg/L), median (IQR)	81 (37–150)	85 (49–159)	78 (32–150)	0.213

Vaccinated: at least once with any COVID-19 vaccine

Analysis: independent T test / Chi square test / Mann Whitney U test

Missing total group: 15 living at home, 64 Katz ADL score, 64 risk of physical impairment, 66 risk of falling, 78 risk of delirium, 103 risk of malnutrition, 57 Clinical Frailty Scale, 18 duration of symptoms, 10 body temperature, 10 respiratory rate, 29 oxygen amount needed, 29 no oxygen needed, 4 C-reactive protein

Missing not vaccinated: 6 living at home, 20 Katz ADL score, 20 risk of physical impairment, 20 risk of falling, 26 risk of delirium, 27 risk of malnutrition, 15 Clinical Frailty Scale, 4 duration of symptoms, 3 body temperature, 1 respiratory rate, 7 oxygen amount needed, 7 no oxygen needed

Missing vaccinated: 9 living at home, 33 smoking, 50 BMI, 44 Katz ADL score, 44 risk of physical impairment, 46 risk of falling, 52 risk of delirium, 76 risk of malnutrition, 42 Clinical Frailty Scale, 14 duration of symptoms, 7 body temperature, 9 respiratory rate, 22 oxygen amount needed, 22 no oxygen needed 4 C-reactive protein

*N* number, *IQR* interquartile range, *SD* standard deviation, *ADL* Activities of Daily Living

^a^COPD, Asthma, interstitial lung disease or lung cancer

^b^Katz ADL score ≥2

In-hospital outcomes are shown in Table 2. Compared to unvaccinated patients, in-hospital mortality was lower in the group of vaccinated patients (22.0% vs. 33.8%; p = 0.037). Hospital length of stay and discharge destination (home, rehabilitation centre or other) were similar in both groups.

**Table 2:** In-hospital outcomes for older hospitalized COVID-19 patients stratified by vaccination status

	Total group	Unvaccinated	Vaccinated	p-value
	N = 327	N = 77	N = 250	
In-hospital mortality, n (%)^a^	81 (24.8)	26 (33.8)	55 (22.0)	0.037
Hospital length of stay (days), median (IQR)	7 (4–13)	7 (4–14)	7 (4–12)	0.891
Discharge destination, n (%)				0.221
Home	181 (73.6)	38 (74.5)	143 (73.3)	
Rehabilitation center	51 (20.7)	12 (23.5)	39 (20.0)	
Other	14 (5.7)	1 (2.0)	13 (6.7)	

Analysis: independent T test/Chi square test/Mann Whitney U test

No missing data

^a^Defined as deceased in hospital and discharge to hospice

Univariable associations of baseline characteristics with in-hospital mortality of all patients are shown in Table 3. Patients of male sex [HR 1.71 (95% CI 1.04–2.081); p = 0.035] and patients with higher respiratory rate (RR) [RR > 20/min, HR 1.71 (95% CI 1.05–2.78); p = 0.032] had a higher risk of death during admission. In this univariable analysis frail patients did not have a significant higher risk for mortality [HR 1.57 (95% CI 0.85–2.89); p = 0.149]. Vaccinated patients had a 35% lower risk of death during admission, although not statistically significant [HR 0.65 (95% CI 0.41–1.04); p = 0.071].

**Table 3:** Univariable Cox regression analysis of in-hospital mortality and patient characteristics per number of admission days for older hospitalized COVID-19 patients

	n/N	HR (95%CI)	p-value
Demographics and comorbidity			
Age (per year)	81/327	1.03 (0.99–1.06)	0.130
Male sex	81/327	1.71 (1.04–2.81)	0.035
Living at home	72/315	0.66 (0.29–1.53)	0.334
Vaccinated	81/327	0.65 (0.41–1.04)	0.071
Charlson Comorbidity Index (per point)	81/327	0.97 (0.87–1.09)	0.648
History of chronic lung disease^a^	81/327	1.39 (0.88–2.20)	0.162
History of dementia	81/327	0.45 (0.11–1.82)	0.260
Geriatric measurements			
Risk of physical impairment^b^	62/266	0.87 (0.51–1.49)	0.621
Risk of falling	60/265	1.07 (0.60–1.93)	0.821
Risk of delirium	57/254	0.75 (0.44–1.29)	0.299
Risk of malnutrition	52/230	0.72 (0.31–1.70)	0.456
Clinical Frailty Scale	64/275		
1–3 (fit)		ref	ref
4–5 (pre-frail)		1.55 (0.81–2.97)	0.190
6–9 (frail)		1.57 (0.85–2.89)	0.149
Disease severity indicators			
Duration of symptoms till admission	79/309		
<5 days		1.35 (0.80–2.27)	0.264
5–7 days		ref	ref
>7 days		0.69 (0.37–1.28)	0.238
Temperature (°C)	79/334		
35.5–38.5		ref	ref
>38.5		0.87 (0.50–1.52)	0.632
Respiratory rate (breaths/min)			
12–20	79/317	ref	ref
>20		1.71 (1.05–2.78)	0.032
Oxygen amount needed (L/min)	76/299		
0		ref	ref
1–5		1.23 (0.59–2.58)	0.571
>5		1.80 (0.85–3.83)	0.125
C-reactive protein (mg/L)	80/323		
<10		ref	ref
10–100		0.85 (0.26–2.80)	0.789
>100		1.50 (0.47–4.85)	0.494

Analysis: Cox regression

*n* number of deceased patients, *N* total number of cases without missing values

*N* number, *IQR* interquartile range, *SD* standard deviation, *ADL* Activities of Daily Living

^a^COPD, Asthma, interstitial lung disease or lung cancer

^b^Katz ADL score ≥ 2

A Cox regression multivariable analysis with different models for the association of vaccination with in-hospital mortality is shown in Table 4. After adjustment for age, male sex and not living at home the risk of death during admission was lower for vaccinated patients [model 1 HR 0.35 (95% CI 0.20–0.61); p ≤ 0.001]. After additional adjustment for high respiratory rate [model 2 HR 0.34 (95% CI 0.19–0.60); p ≤ 0.001] and for high CFS (CFS ≥ 4) and CCI the risk of death in hospital was three times lower for vaccinated patients [model 3 HR 0.30 (95% CI 0.16–0.56); p < 0.001]. A Cox regression multivariable analysis for the association of vaccination with in-hospital mortality for frail patients only (CFS ≥ 4) is shown in Table 5. After adjustment for the same risk factors as in Table 4, there is a comparable significantly lowered risk of in-hospital death [model 3 HR 0.32 (95% CI 0.13–0.79); p 0.013].

**Table 4:** Multivariable Cox regression analysis of vaccination effect on in-hospital mortality for older hospitalized COVID-19 patients

	n/N	HR (95%CI)	p-value
Crude			
Vaccinated	81/327	0.65 (0.41–1.04)	0.071
Model 1			
Vaccinated, age (per year), male sex, not living at home	72/315	0.35 (0.20–0.61)	<0.001
Model 2			
Vaccinated, age (per year), male sex, not living at home, high respiratory rate^1^	70/305	0.34 (0.19–0.60)	<0.001
Model 3			
Vaccinated, age (per year), male sex, not living at home, high respiratory rate^1^, high CFS^2^ and CCI (per point)	59/260	0.30 (0.16–0.56)	<0.001

*n* number of deceased patients, *N *total number of cases without missing values, *CFS* Clinical Frailty Scale, *CCI* Charlson Comorbidity index

^1^Respiratory rate > 20/min, ^2^CFS ≥ 4

Missings: 12 living at home, 10 respiratory rate, 52 CFS

**Table 5:** Multivariable Cox regression analysis of vaccination effect on in-hospital mortality for older frail^1^ hospitalized COVID-19 patients

	n/N	HR (95%CI)	p-value
Crude			
Vaccinated	46/176	0.55 (0.29–1.05)	0.071
Model 1			
Vaccinated, age (per year), male sex, not living at home	44/173	0.32 (0.15–0.67)	0.003
Model 2			
Vaccinated, age (per year), male sex, not living at home, high respiratory rate^1^	42/166	0.31 (0.14–0.66)	0.003
Model 3			
Vaccinated, age (per year), male sex, not living at home, high respiratory rate^1^, high CFS^2^ and CCI (per point)	29/127	0.32 (0.13–0.79)	0.013

Missings: 12 living at home, 10 respiratory rate, 52 CFS

*n* number of deceased patients, *N* total number of cases without missing values, *CFS* Clinical Frailty Scale, *CCI* Charlson Comorbidity index

^1^Respiratory rate > 20/min, ^2^CFS ≥ 4

## Discussion

The main findings of this study were twofold. First, vaccinated patients were older, had more comorbidities and were frailer at hospitalization for COVID-19 infection. Second, after correction for pre-existing mortality risk factors, risk of in-hospital mortality was three times lower for vaccinated patients.

At hospitalization for COVID-19, vaccinated patients were older and more frequently male, which is in accordance with another Dutch COVID-19 study comparing vaccinated and unvaccinated in-hospital patients of all ages [15]. In addition, vaccinated patients were frailer, had more comorbidities and were less often living at home. Vaccinated patients were hospitalized in an earlier stage of disease, although they were equally ill to unvaccinated patients at time of hospital admission. In a previous study describing Winter 2020, before the vaccination program started, patients, frail and fit, have been admitted after a median of 6 symptomatic days (IQR 3–9) [6], exactly equal to the number of symptomatic days before admission for the total group in the present study. It is unknown whether unvaccinated patients waited longer to be hospitalized because of better physical reserves or other reasons. Presumably, many fit unvaccinated and fit vaccinated patients did not need hospitalization at all and therefore the present patient population is a specific selection from older people in the community. It is known that frail older people have less physical reserve and for that reason need hospitalization for acute illness more often than fit older people [16]. Therefore, the earlier admission of vaccinated patients may be explained by the effect of frailty, dominantly present in this group. In fact, other mentioned differences between vaccinated and unvaccinated patients are probably all related to more frailty in the vaccinated group.

The overall in-hospital mortality was 24.8%. This is comparable to the in-hospital mortality among older hospitalized COVID-19 patients in a previous study in The Netherlands in Winter 2020 (26%) [6]. Since then, there have not been any significant changes in medical treatment and diagnostics for COVID-19 infection in The Netherlands [8]. However, vaccinated hospitalized patients in this present study were older, frailer and more had more comorbidities, which would have resulted in a higher mortality risk from the beginning of hospital admission. A recent review states that frailty leads to an elevated risk of mortality for COVID-19 and this risk can be aggravated by other risk factors such as comorbidities [17]. Besides, we do know that vaccination may lead to lower risk of short term mortality after hospitalisation; a study on hospitalized COVID-19 patients of all ages showed that unvaccinated patients had a higher risk of 28-day mortality (HR 2.25 95% CI 1.18–4.27) adjusted for age, sex and immune status [15]. However, in this present study, after correction for pre-existing mortality risk factors, risk of in-hospital mortality was three times lower for vaccinated patients. In conclusion, vaccinated patients benefit from the protective effect of the vaccine against death during their hospital stay, overruling their increased chance of dying because of being older, frailer and having more comorbidities.

This study demonstrated the possible benefits of COVID-19 vaccination for older and especially frail patients in the hospital. With the introduction of COVID-19 vaccination, characteristics and outcomes of the in-hospital older COVID-19 patient population changed and may necessitate different healthcare needs. For instance, the older and frailer vaccinated population has a fair chance of surviving hospital admission, which may trigger pro-active geriatric advance care planning, aimed towards early rehabilitation. Finally, the study results can support clinical insight in the prognosis for an individual older patient admitted for COVID-19 infection. In the NICE COVID-19 guideline [14] the CFS is recommended to be used to assess frailty. Although in earlier guideline versions the CFS was recommended to assess risk of mortality and decision making on therapeutic limitations, the most recent guideline version recommends a holistic assessment. Our study supports the statement that assessing frailty only is not enough to estimate mortality risk for older hospitalized patients with acute disease. It might be that the effect of frailty can even be diminished by other distinctive factors such as vaccination.

Our study had a few limitations. The study population was small, although all COVID-19 patients aged 70 and older admitted to the clinical wards of these 5 hospitals have been included in the database. While one should be careful interpreting the results, the study population is a representative sample of older patients admitted with COVID-19 infection in academic and non-academic hospitals in the Netherlands. CFS was scored retrospectively for part of the study population, which could have led to less precise classification of individuals. However, retrospective attainment of CFS scores has been validated and is considered to be as reliable and accurate as prospective attainment [18]. The study also has several strengths. A wide variety of variables had been collected including demographics, comorbidity, frailty, disease severity indicators and discharge destination. Patients admitted for COVID-19 infection had been selected and patients admitted for another reason had been excluded in order to select a representative group. The effect of vaccination on in-hospital mortality may have been influenced by confounders, therefore we included possible confounders in a multivariable analysis.

In conclusion, compared to older unvaccinated patients hospitalized for COVID-19 infection, vaccinated patients were older, frailer, had more comorbidities and, independent of these pre-existing mortality risk factors, a three times lower risk for in-hospital mortality. These findings suggest that vaccinated patients benefit from the protective effect of the vaccine against death during hospital stay, outweighing the increased mortality risk that is associated with older age, greater frailty and more numerous comorbidities. This could be an encouragement for older people to receive age-appropriate vaccines, although no definite conclusions can be drawn, for this was no intervention study. The results may trigger pro-active geriatric advance care planning, aimed towards early rehabilitation of the survivors and also suggest that assessing frailty only is not enough to estimate mortality risk for older hospitalized patients with acute disease.

## Data Availability

The data is available upon request, the PI can be contacted for more information.

## References

[1] Haas EJ et al (2021) Impact and effectiveness of mRNA BNT162b2 vaccine against SARS-CoV-2 infections and COVID-19 cases, hospitalisations, and deaths following a nationwide vaccination campaign in Israel: an observational study using national surveillance data. Lancet 397(10287):1819–1829 33964222 10.1016/S0140-6736(21)00947-8 PMC8099315

[2] Pastorino R et al (2022) Change in age distribution of COVID-19 deaths with the introduction of COVID-19 vaccination. Environ Res 204(Pt C):112342 34748775 10.1016/j.envres.2021.112342 PMC8570444

[3] Chen CL et al (2024) Clinical characteristics and treatment outcomes among the hospitalized elderly patients with COVID-19 during the late pandemic phase in central Taiwan. J Microbiol Immunol Infect 57(2):257–268 38326193 10.1016/j.jmii.2024.01.006

[4] RIVM (2024) Figues on the COVID-19 vaccination programme. https://www.rivm.nl/en/coronavirus-covid-19/current/vaccination-figures. Accessed 17 Jan 2024

[5] Blomaard LC et al (2021) Frailty is associated with in-hospital mortality in older hospitalised COVID-19 patients in the Netherlands: the COVID-OLD study. Age Ageing 50(3):631–640 33951156 10.1093/ageing/afab018 PMC7929372

[6] Smits RAL et al (2022) Characteristics and outcomes of older patients hospitalised for COVID-19 in the first and second wave of the pandemic in The Netherlands: the COVID-OLD study. Age Ageing 51(3):afac048 35235650 10.1093/ageing/afac048 PMC8890695

[7] team tN (2022) Genomic epidemiology of SARS-CoV-2 with subsampling focused on Europe since pandemic start. https://nextstrain.org/ncov/open/europe/all-time. Accessed 5 May 2024

[8] Organisation WH (2022) Therapeutics and COVID-19: living guideline. https://app.magicapp.org/#/guideline/nBkO1E/rec/nBMO8R. Accessed 5 May 2024

[9] Charlson ME et al (1987) A new method of classifying prognostic comorbidity in longitudinal studies: development and validation. J Chronic Dis 40(5):373–383 3558716 10.1016/0021-9681(87)90171-8

[10] VMS (2009) Praktijkgids ' Kwetsbare Ouderen'. Den Haag: VMS Veiligheidsprogramma. https://www.vmszorg.nl/wp-content/uploads/2017/11/web_2009.0104_praktijkgids_kwetsbare_ouderen.pdf. Accessed 5 May 2024

[11] Katz S et al (1963) Studies of illness in the aged. The index of Adl: a standardized measure of biological and psychosocial function. JAMA 185:914–9 14044222 10.1001/jama.1963.03060120024016

[12] Kruizenga HM et al (2005) Development and validation of a hospital screening tool for malnutrition: the short nutritional assessment questionnaire (SNAQ). Clin Nutr 24(1):75–82 15681104 10.1016/j.clnu.2004.07.015

[13] Rockwood K et al (2005) A global clinical measure of fitness and frailty in elderly people. CMAJ 173(5):489–495 16129869 10.1503/cmaj.050051 PMC1188185

[14] NICE (2021) COVID-19 rapid guideline: managing COVID-19. https://www.nice.org.uk/guidance/ng191. Accessed 25 Jan 2024

[15] Leuning DG et al (2022) Significant impact of vaccination on length of hospital stay and survival in hospitalized patients with COVID-19. New Microbes New Infect 49:101047 36440092 10.1016/j.nmni.2022.101047 PMC9678229

[16] Blomaard LC et al (2020) Geriatric screening, triage urgency, and 30-day mortality in older emergency department patients. J Am Geriatr Soc 68(8):1755–1762 32246476 10.1111/jgs.16427 PMC7497167

[17] Tana C et al (2023) Approach to COVID-19 in older adults and indications for improving the outcomes. Ann Med 55(2):2265298 37839411 10.1080/07853890.2023.2265298 PMC10578089

[18] Stille K et al (2020) Validation of the Clinical Frailty Scale for retrospective use in acute care. Eur Geriatr Med 11(6):1009–1015 32770462 10.1007/s41999-020-00370-7

